# An alternative renin isoform is cardioprotective by modulating mitochondrial metabolism

**DOI:** 10.1111/jcmm.13872

**Published:** 2018-09-24

**Authors:** Heike Wanka, Philipp Lutze, Doreen Staar, Bianka Grunow, Barbara S. Peters, Jörg Peters

**Affiliations:** ^1^ Department of Physiology University Medicine of Greifswald Karlsburg Germany

**Keywords:** aerobic glycolysis, cytosolic renin, exon(1A‐9)renin, exon‐b renin, H9c2 cells, heart, mitochondrial spare capacity, Warburg effect

## Abstract

The renin‐angiotensin system promotes oxidative stress, apoptosis, necrosis, fibrosis, and thus heart failure. Secretory renin plays a central role in these processes, initiating the generation of angiotensins. Nevertheless, alternative renin transcripts exist, which code for a cytosolically localized renin isoform (cyto‐renin) that is cardioprotective. We tested the hypothesis that the protective effects are associated with a beneficial switch of metabolic and mitochondrial functions. To assess H9c2 cell mitochondrial parameters, we used the Seahorse XF analyser. Cardiac H9c2 cells overexpressing cyto‐renin exhibited enhanced nonmitochondrial oxygen consumption, lactate accumulation, and LDH activity, reflecting a switch to more aerobic glycolysis known as Warburg effect. Additionally, mitochondrial spare capacity and cell respiratory control ratio were enhanced, indicating an increased potential to tolerate stress conditions. Renin knockdown induced opposite effects on mitochondrial functions without influencing metabolic parameters. Thus, the protective effects of cyto‐renin are associated with an altered bioenergetic profile and an enhanced stress tolerance, which are favourable under ischaemic conditions. Therefore, cyto‐renin is a promising new target for the prevention of ischaemia‐induced myocardial damage.

## 1 INTRODUCTION

The renin‐angiotensin system (RAS) promotes hypertrophy, increases oxidative stress, and exerts pro‐inflammatory effects in the heart. Renin is a secretory glycoprotein that generates angiotensin (ANG) I from its only known substrate, angiotensinogen. ANG I is then cleaved to ANG II, the effector peptide of the RAS. ANG II leads to apoptosis, necrosis, fibrosis, myocardial remodelling, and thus promotes cardiac failure. Correspondingly, inhibitors of the RAS are among the most potent drugs in the treatment of hypertension and cardiac failure, markedly increasing the life span of patients.^1^

In addition to the classical renin transcript, alternative transcripts have been identified in several species including humans [termed exon(2‐9), exon(1A‐9), renin‐b, renin‐c].^2^, ^3^, ^4^ These alternative transcripts are derived from the same renin gene, but lack exon 1. Exon 1 encodes for a signal sequence required for the cotranslational transport to the endoplasmatic reticulum (ER), and thus for the sorting of renin to the secretory pathway. All alternative renin transcripts are translated into a truncated prorenin. The protein, which we henceforth term cyto‐renin, lacks the prefragment and the first 15 amino acids of the conventional prorenin.^2^, ^3^, ^4^ Cyto‐renin is also localized within mitochondria.^3^, ^5^, ^6^

The transcription of cyto‐renin is under the control of an alternative promoter located in intron 1.^7^ This promoter has very low basal activity, but is stimulated by glucose depletion in cardiac cells^7^ as well as after myocardial infarction in the rat heart.^8^ Cells overexpressing cyto‐renin are more resistant against necrotic death under ischaemic conditions such as glucose depletion.^9^ Moreover, overexpression of cyto‐renin in the heart of transgenic rats reduces infarct size in ex vivo Langendorff preparations.^9^

In the present study, we tested the hypothesis that cyto‐renin overexpression exerts favourable effects on metabolic and mitochondrial functions, which may explain the reduced infarct size ex vivo or the protective effects in vitro. The investigations are based on measurements of oxygen consumption (OCR) and extracellular acidification rate (ECAR) using the Seahorse technology^10^, ^11^ as well as LDH activity, extracellular glucose and lactate contents. Thus, we were able to assess the impact of cyto‐renin on the bioenergetic profile of H9c2 cardiomyoblasts. Specifically, we analysed the ratio between mitochondrial vs nonmitochondrial respiration, respiratory‐dependent ATP synthesis, proton leak, and spare respiratory capacity after addition of specific inhibitors of the respiratory chain.

## 2 METHODS

### 2.1 Cell culture

The H9c2 cell line obtained from American Tissue Type Collection (Manassas, VA, USA) was cultured in DMEM medium supplemented with 100 U/mL penicillin, 100 μg/mL streptomycin and 10% foetal bovine serum in 75 cm^2^ tissue culture flasks at 37°C in a humidified atmosphere of 5% CO_2_. Cells were fed every 3 days and subcultured after reaching around 80% confluence. H9c2 cells were transfected with a pIRES/Neo vector ± ren(2‐9) cDNA as previously described.^6^ Upregulation of ren(2‐9) was 20‐fold as determined by qRT‐PCR analysis using the renin forward primer *GCTCCTGGCAGATCACCAT and the* reverse primer *CCTGGCTACAGTTCACAACGTA*.

Downregulation of renin in H9c2 cells was accomplished with the RNA interference method using 80 nmol/L siGENOME SMART pool siRNA to renin (Dharmacon, Thermo Fisher Scientific, Schwerte, Germany) according to the manufacturer's instructions. OptiMEM (Gibco, Thermo Fisher Scientific, Schwerte, Germany) served as transfection medium and 4 μL of a polyethyleneimine solution (1 μg/μL) (Sigma‐Aldrich, Taufkirchen, Germany) as transfection reagent. SiRNA‐mediated knockdown was performed for 24 hours. Renin transcript abundance was quantified by qRTPCR analysis using the ren(1‐9) primer pairs *for*: ATG*AATTCACCCCATTCAGC* and rev: *CCAGATGGGCGGGAGGAGGATG,* and the ren(1A‐9) primer pairs for: *TGAATTTCCCCAGTCAGTGAT* and rev: *GAATTCACCCCATTCAGCAC*.

### 2.2 Extracellular glucose and lactate levels

After 24 hours incubation of untreated H9c2 cells, ren(2‐9) cells or siRNA pretreated cells (scramble vrs. siRNA to renin) in 2 mL DMEM medium at 37°C, glucose and lactate levels of the cell culture supernatant were determined using the Biosens C line GF+ analyser (EKF Diagnostics, Germany). Glucose and lactate levels of the medium incubated without cells were measured simultaneously to calculate glucose consumption and lactate accumulation rates. An amount of 20 μL of the culture supernatants was incubated with 1 mL haemolysing solution using prefilled safe‐lock reaction cups (EKF Diagnostics, Germany). Glucose consumption and lactate accumulation, respectively, were normalized to the cell numbers.

### 2.3 LDH activity

Lactate dehydrogenase (LDH) activity was determined using the Cytotoxicity Detection Kit (Roche Applied Science, Mannheim, Germany) with slight modifications. Before measurements were performed according to manufacturer's instructions, 10 000 cells in 100 μL medium were seeded in 96‐well plates and incubated for 24 hours at 37°C in a CO_2_ incubator. Then, the cells were lysed by addition of 1% triton X‐100 for 1 hour to detect total LDH activity.

### 2.4 Seahorse XF analysis

The Seahorse XF analyser (Agilent Technologies, Seahorse Bioscience, Santa Clara, USA) was used to assess H9c2 extracellular acidification rate (ECAR, mpH/min) and cell mitochondrial oxygen consumption rate (OCR, pmol/min), including basal mitochondrial and nonmitochondrial OCRs, maximal respiration, proton leak, spare respiratory capacity, and ATP production. To determine optimal cell density for the experiments, different H9c2 cell numbers, ranging from 5000 to 180 000 cells/well, were seeded into the wells of a XF96‐well plate (Agilent Technologies, Seahorse Bioscience, Santa Clara, USA). Subsequent experiments were started with 20 000 cells/well.

H9c2 cells were seeded in 200 μL DMEM medium per well in XF96‐well plates and cultured overnight in a CO_2_ incubator. Culture medium was replaced by 180 μL modified, pH‐controlled XF base medium (Agilent Technologies, Seahorse Bioscience) without HCO_3_^−^ supplemented with 10 mmol/L glucose, 2 mmol/L sodium pyruvate, and 2 mmol/L glutamine. Then, cells were placed in a non‐CO_2_ incubator at 37°C for 1 hour. After preparation and application of compounds of the XF Cell Mito Stress Test Kit (Agilent Technologies, Seahorse Bioscience, Santa Clara, USA) into cartridge ports, the cartridge and subsequently the cell culture plate were loaded into the XF96 Seahorse analyser. Following an equilibration period, different running cycles (each mix, wait, measure) were performed. After three recording cycles to determine the basal OCR, 20 μL oligomycin (1 μmol/L final concentration) were injected into the wells to inhibit ATP synthase, thereby calculating the proportion of respiration used to drive ATP synthesis. After three further cycles, 22 μL of carbonyl cyanide‐4‐(trifluoromethoxy) phenylhydrazone (FCCP; 1 μmol/L) was injected to uncouple ATP synthesis from electron transport to determine maximal mitochondrial respiration. Finally, 25 μL of a solution containing the inhibitors of the respiratory chain complexes I and III, rotenone and antimycin A (0.5 μmol/L each), were injected for another three cycles to determine the nonmitochondrial respiration rate.

Based on the basal parameters, we determined the coupling efficiency of oxidative phosphorylation (CE) and the maximal cellular respiratory control (CRC_max_).^10^ CE was calculated as oligomycin‐sensitive portion of mitochondrial respiration. CRC was derived from the ratio of FCCP‐stimulated maximal respiration and oligomycin‐insensitive proton leak OCR. These parameters reflect electron transport and/or substrate oxidation as well as mitochondrial quality.

### 2.5 Measurement of protein content

To control for variations in cell number due to different growth rates between H9c2 controls and ren(2‐9)‐expressing cells, we determined the protein contents per well after analysis of Seahorse experiments. After completion of the assay, the media of the cell plate was carefully aspirated and the remaining cells were lysed in 12 μL RIPA buffer containing 33.3 mmol/L Tris, 3.33 mmol/L EDTA, 100 mmol/L NaCl, 6.67 mmol/L K_2_HPO_4_, 6.67% glycerol, 0.67% TritonX‐100, 1 mmol/L NaVO_4_, 20 mmol/L NaF, 0.1 mmol/L PMSF, 20 mmol/L 2‐phosphoglycerat and a protease inhibitor cocktail (Roche Diagnostics, Mannheim, Germany). An amount of 50 μL of the Bradford reagent (Roth, Karlsruhe, Germany) was then added to 50 μL of the 1:10 diluted probe. Protein content was measured according to the manufacturer's instructions.

### 2.6 Determination of mitochondrial DNA content

After isolation of genomic DNA using the innuPREP DNA Mini Kit (analyik Jena, Germany) according to manufacturer's instruction, the mitochondrial content of H9c2, pIRES control cells and ren(2‐9)‐expressing cells was determined by the Rat Mitochondrial DNA Copy Number Assay Kit (Detroit R&D, Inc., USA). The assay based on the comparison of mitochondrial to nuclear DNA content using validated primers and real‐time PCR. The calculation of mitochondrial copy number was performed by analysing the cycle numbers (Ct) according to the formula: ∆Ct = −Ct (mitochondrial‐control)−Ct (nucleus‐control).

### 2.7 Flow cytometry and microscopic imaging of mitochondrial parameters

Mitochondrial membrane potential (∆Ψ_m_) is an indicator of mitochondrial state. It was measured in H9c2 control lines and ren(2‐9)‐expressing cells using 5,5′,6,6′‐tetrachloro‐1,1′,3,3′‐tetramethylbenzimidazolylcarbocyanine iodide (JC1) or MitoTracker red CMXRos according to manufacturer's instructions (Molecular Probes, Thermo Fisher Scientific, Germany). 1 x 10^5^ cells in 0.5 mL culture DMEM were incubated with either 4 μmol/L of JC1 solution for 15 minutes or with 100 nmol/L MitoTracker red CMXRos for 45 minutes at 37°C in the CO_2_ incubator. For assessment of JC1 and MitoTracker staining, a total of 5000 gated events were analysed using the flow cytometer (FACS Calibur, BD, Franklin Lakes, NJ, USA). For JC1 excitation, a 488 nm filter was used. Emission filters of 535 nm and 595 nm were used to quantify cells with green (JC1 monomers) and orange (JC1 aggregates) fluorescence, respectively. The ratio of orange/green fluorescence intensity is determined by the mitochondrial membrane potential.

For microscopy, control and ren(2‐9)‐overexpressing cells were plated at a density of 20 000 cells/well in Lab‐Tec four‐chamber cover glass slides (Thermo Fisher Scientific, Schwerte, Germany) and preincubated for 3 days. After exchange of the medium, cells were incubated either with 1 μmol/L MitoTracker red CMXRos or 0.1 μmol/L nonyl‐acridine orange in DMEM for 45 minutes at 37°C to detect the mitochondrial network. Following removal of medium and three washings in Hank's balance salt solution (HBSS), cells were fixed in 2% paraformaldehyde for 10 minutes at room temperature (RT). Fixing solution was removed by washing the cells in HBSS. To detect the cellular localization of mitochondria, membranes were labelled by application of a rabbit anti‐mouse Alexa‐488 conjugated wheat germ agglutinin (WGA) antibody for 30 minutes at room temperature (1:500 diluted, Thermo Fisher Scientific, Germany). To label the nucleus, cells were finally incubated with DAPI (DAKO Omnis, Hamburg, Germany) for 5 minutes at RT washed with H_2_O_dest_ and mounted onto glass slides for 24 hours at 4°C. Slides were imaged with a 100x objective using the fluorescence microscope BZ II 9000 (Keyence Corp, Osaka, Japan). Additionally, we used the line profile analyser (BZ II software) to estimate the location of the mitochondria between the nucleus and the cell membrane.

### 2.8 Statistical analyses

The data presented are means ± SEM of independently performed experiments. Each of the Seahorse experiments comprises data of three wells per group. One‐way ANOVA or two‐way ANOVA with Bonferroni posttest analyses were performed as appropriate using GraphPad Prism (Graph Pad Software, La Jolla, CA, USA). Values of *P* < 0.05 were considered statistically significant.

## 3 RESULTS

### 3.1 Nonmitochondrial O_2_ consumption is increased in ren(2‐9) cells

**Figure 1:**
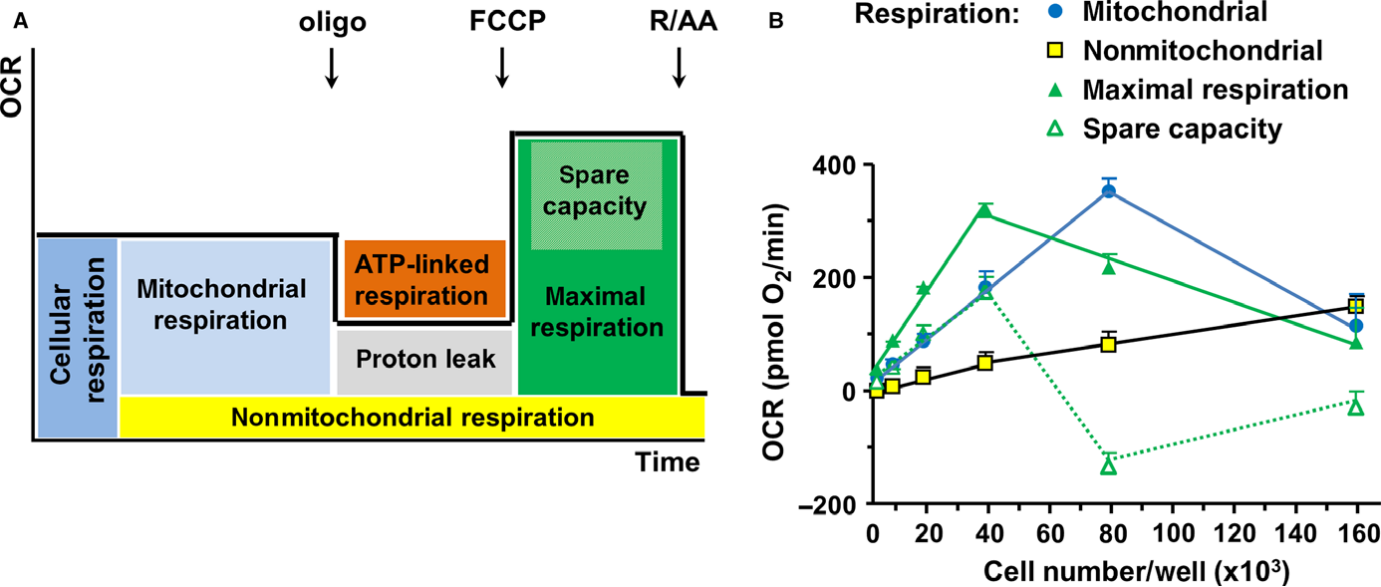
Basal oxygen consumption rate increases with cell number. Oxygen consumption rate (OCR) was analysed using the Seahorse technology. Real‐time measurements were obtained from H9c2 cells seeded at different numbers per well (n = 7). A, Distinct modules of cellular oxygen consuming processes. B, Real time OCRs obtained from H9c2 cells seeded with different cell numbers/well (n=6). Data represent mean ± SEM values

We used H9c2 cardiomyoblasts as an alternative model to study mitochondrial functions because unfortunately, the Seahorse system does not yield reliable results with primary adult rat cardiomyocytes (own experience and personal communication with staff of Agilent Technologies, Seahorse Bioscience, Santa Clara, USA). The time‐lapse measurements of respiration show distinct components of oxygen consumption, reflecting the different mitochondrial and cellular processes as indicated in Figure 1A. First, we optimized the culture conditions in relation to the cell number seeded per well. The data reveal a strong dependency of the distinct oxygen consuming processes from the cell number (Figure 1B). Total cellular oxygen consumption rate (OCR) comprises mitochondrial and nonmitochondrial respiration, which can be differentiated by blocking the mitochondrial respiratory chain with rotenone and antimycin A. While nonmitochondrial OCR increased with seeding density, mitochondrial OCR increased linearly only in a range of 5 000‐80 000 cells per well. A further doubling of cell number was accompanied by a marked decline of OCR reflecting a reduced viability of the cells. The consequences of enhanced seeding cell number were even much more pronounced considering the maximal respiration which is detectable after uncoupling of respiratory chain by FCCP. Maximal OCR markedly decreased already at a cell number higher than 40 000 per well. Subsequently, spare respiratory capacity obtained by subtracting mitochondrial OCR from FCCP‐induced maximal respiration was lost at that cell density. Therefore, subsequent experiments were started with a cell number of 20 000 cells/well to remain in the linear range if a prolongation of preculture time associated with an increase of cell number was necessary for instance in the knockdown experiments.

**Figure 2:**
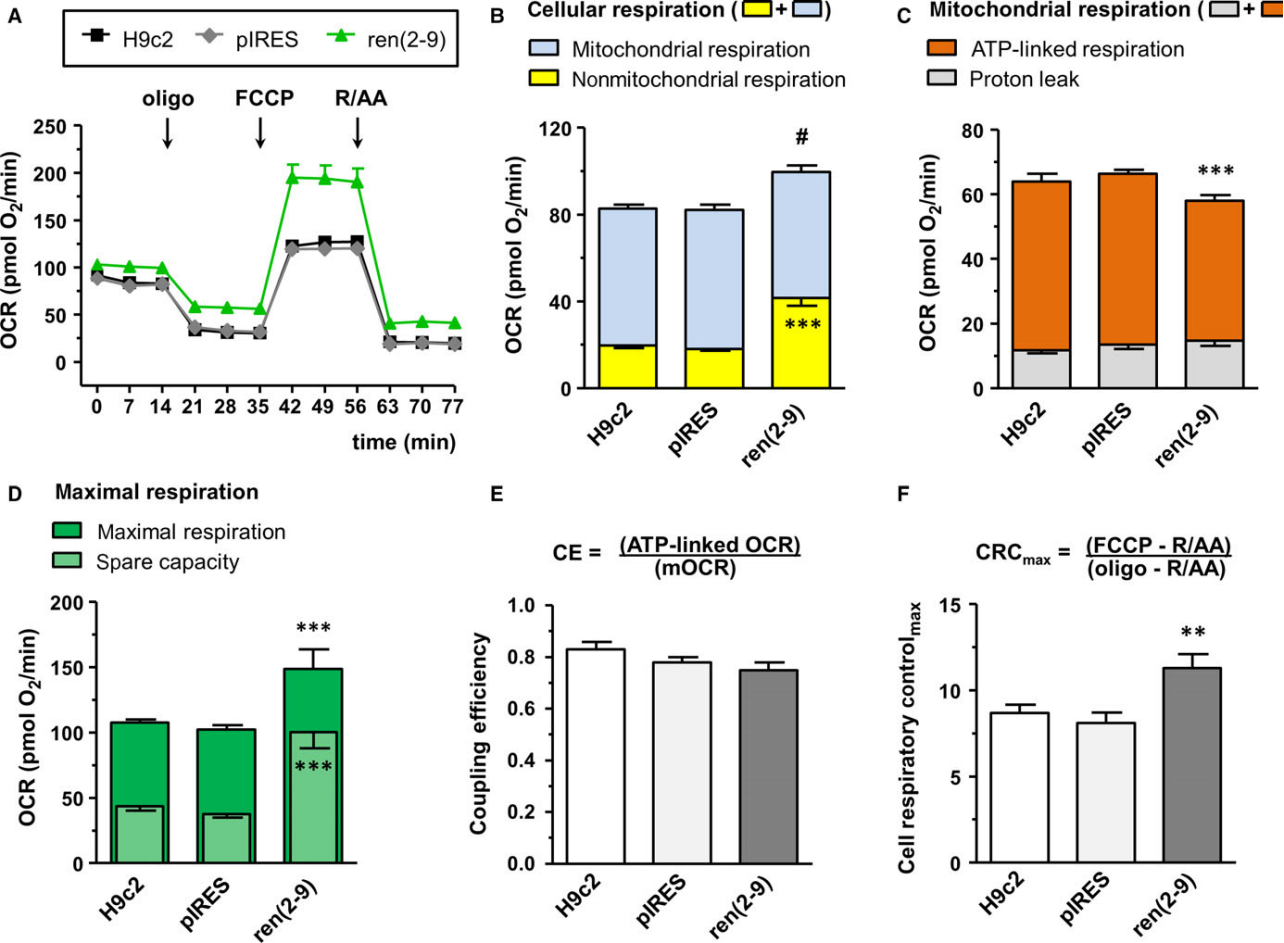
Mitochondria of cyto‐renin overexpressing cells exhibit a beneficial bioenergetic profile. Oxygen consumption rate (OCR) was analysed after injection of inhibitors of the respiratory chain at indicated time‐points. A, Real‐time OCRs obtained from H9c2 cells (n = 11), pIRES control cells (n = 12) and ren(2‐9) cells (n = 20). B, OCRs in cells after injection of rotenone (R) and antimycin A (AA) (cellular respiration) according to mitochondrial (blue) and nonmitochondrial components (yellow). C, OCRs in mitochondria after injection of oligomycin (Oligo) (mitochondrial respiration) according to ATP‐linked respiration (orange) and proton leak (grey). D, Maximal OCRs obtained after injection of the uncoupler FCCP (maximal respiration) (green) and spare capacity (green shaded). Spare capacity was calculated by subtracting mitochondrial respiration from maximal respiration. E, Coupling efficiency (CE) of oxidative phosphorylation was determined as the oligomycin‐sensitive portion of mitochondrial respiration. F, Maximal cell respiratory control (CRC_Max_) was derived from the ratio of maximal respiration to oligomycin‐insensitive proton leak OCR. Data represent mean ± SEM values with ***P < 0.001 or **P < 0.01 control cell lines vs ren(2‐9) cells; #*P* < 0.05 total cellular respiration control cell lines vs the ren(2‐9) cells

**Figure 3:**
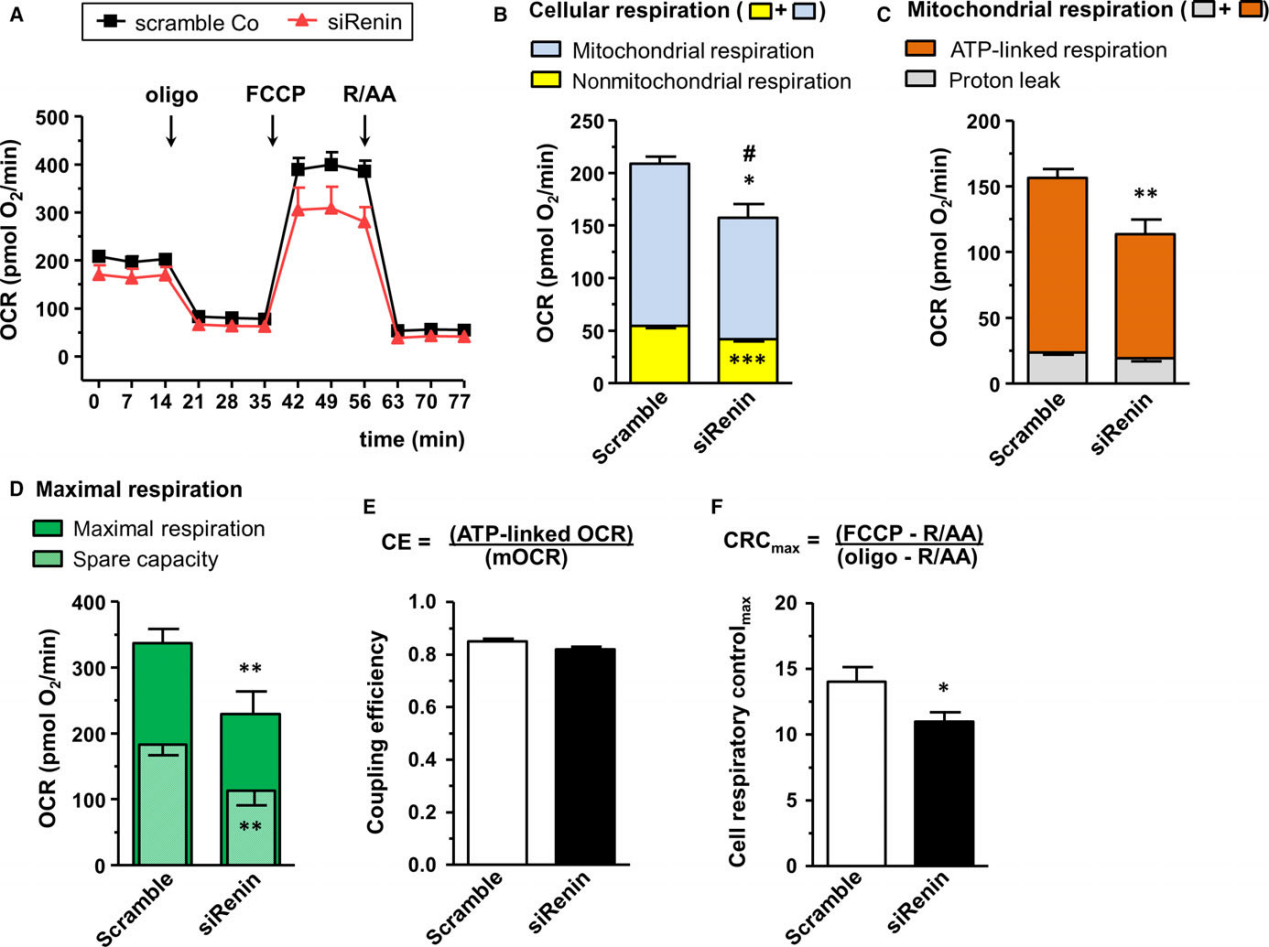
Bioenergetic profile of H9c2 cells is affected disadvantageously by renin knockdown. OCR was analysed after injection of inhibitors of the respiratory chain at indicated time‐points. A, Real‐time OCRs obtained from scramble controls (n = 9) and cells after knockdown of renin using 80 nmol siRNA (n = 9). B, Cellular respiration after injection of rotenone (R) and antimycin A (AA) representing mitochondrial (blue) and nonmitochondrial components (yellow). C, OCRs in mitochondria after injection of oligomycin (Oligo) (mitochondrial respiration) according to ATP‐linked respiration (orange) and proton leak (grey). D, Maximal OCRs (green) measured after injection of the uncoupler FCCP and spare capacity (green shaded). Spare capacity was calculated by subtracting mitochondrial respiration from maximal respiration. E, Coupling efficiency (CE) of oxidative phosphorylation was determined as the oligomycin‐sensitive portion of mitochondrial respiration. F, Maximal cell respiratory control (CRC_Max_) was derived from the ratio of maximal respiration to oligomycin‐insensitive proton leak OCR. Data represent mean ± SEM values with **P < 0.01 or *P < 0.05 scramble control vs siRenin‐treated H9c2 cells. #*P* < 0.05 total cellular respiration scramble control vs siRenin‐treated cells

The time‐lapse measurements of respiration show distinct courses in ren(2‐9) cells compared to H9c2 and pIRES controls (Figure 2A). Total cellular respiration was increased in ren(2‐9) cells (99.6 ± 3.6 pmol O_2_/min) compared to control cell lines H9c2 (82.9 ± 1.9 pmol O_2_/min) and pIRES (82.4 ± 2.3 pmol O_2_/min) (Figure 2B). While mitochondrial OCR as part of cellular respiration was similar in all cell lines, nonmitochondrial OCR was higher in ren(2‐9) cells (41.7 ± 3.6 pmol O_2_/min) than in controls (H9c2: 19.7 ± 1.1 and pIRES: 18.1 ± 1.2 pmol O_2_/min) (Figure 2B). Thus, percentage of nonmitochondrial oxygen consumption in comparison to total respiration amounted to 41.14 ± 2.78% in ren(2‐9) cells compared to 23.85 ± 1.45% and 22.95 ± 1.47% in H9c2 and pIRES cells, respectively. Knockdown of renin induced opposite effects (Figure 3). Total cellular respiration decreased significantly from 202.5 ± 9.6 pmol O_2_/min in scramble controls to 164.3 ± 14.2 pmol O_2_/min in siRenin‐treated H9c2 cells. This decrease was attributed to the significant decline of both mitochondrial as well as nonmitochondrial OCRs (Figure 3B).

To analyse mitochondrial OCRs (mOCRs) in more detail, the ATP synthase inhibitor, oligomycin, was added to the cells. Under these conditions, mOCRs relate to the oxygen consumption driving the mitochondrial proton leak (oligomycin‐insensitive fraction) and ATP‐linked respiration (oligomycin‐sensitive fraction). Levels of mitochondrial proton leak were similar in all cell lines investigated (Figure 2C). In contrast, the ATP‐linked respiration that reveals ATP turnover was reduced in ren(2‐9) cells (43.3 ± 1.9 pmol O_2_/min) compared to control H9c2 and pIRES cells (52.3 ± 2.3 and 52.8 ± 1.3 pmol O_2_/min, respectively) (Figure 2C). Coupling efficiency (CE) of oxidative phosphorylation derived from the proportion of oligomycin‐sensitive OCR to total mOCR however, showed no significant differences between ren(2‐9) cells, H9c2 cells and pIRES cells (0.75 ± 0.03 vs 0.83 ± 0.03 and 0.78 ± 0.02) (Figure 2E). In contrast, downregulation of renin resulted in a significant decrease of ATP‐linked respiration (133.2 ± 0.03 to 94.4 ± 11.0 pmol O_2_/min) whereas proton leak‐associated respiration (Figure 3C) and coupling efficiency were not impaired (Figure 3E).

### 3.2 Ren(2‐9) mitochondria exhibit enhanced spare respiratory capacity and cell respiratory control ratio_max_

Spare respiratory capacity (SRC) represents the ability of the mitochondria to increase respiration to a maximal level when oxidative phosphorylation (OXPHOS) is uncoupled from ATP synthesis by FCCP. The OCRs obtained after FCCP injection showed that maximal respiration was increased in ren(2‐9) cells (148.8 ± 14.8 pmol O_2_/min) compared to H9c2 and pIRES controls (107.5 ± 2.5 and 102.4 ± 3.2 pmol O_2_/min) (Figure 2A, D). SRC obtained by subtracting mitochondrial OCR from FCCP‐induced maximal respiration was about twice as high in ren(2‐9) cells (90.8 ± 12.2 pmol O_2_/min) than in H9c2 and pIRES cells (44.3 ± 2.2 and 38.2 ± 2.1 pmol O_2_/min) (Figure 2D). The ratio between maximal FCCP‐induced OCR and oligomycin‐insensitive respiration, defined as maximal cell respiratory control (CRC_max_), was enhanced in ren(2‐9) cells (11.3 ± 0.8) compared to H9c2 and pIRES cells (8.7 ± 0.5 and 8.1 ± 0.6) (Figure 2F).

Knockdown of renin resulted in opposite responses. Maximal respiration (337.3 ± 21.0 pmol O_2_/min), SRC (183.2 ± 14.5 pmol O_2_/min) (Figure 3D), and CRC_max_ (14.0 ± 1.1) (Figure 3F) decreased significantly to 229.2 ± 34.6 pmol O_2_/min and 114.1 ± 21.6 pmol O_2_/min as well as 10.9 ± 0.7.

### 3.3 Ren(2‐9) expression is associated with an increase of MitoTracker intensity

**Figure 4:**
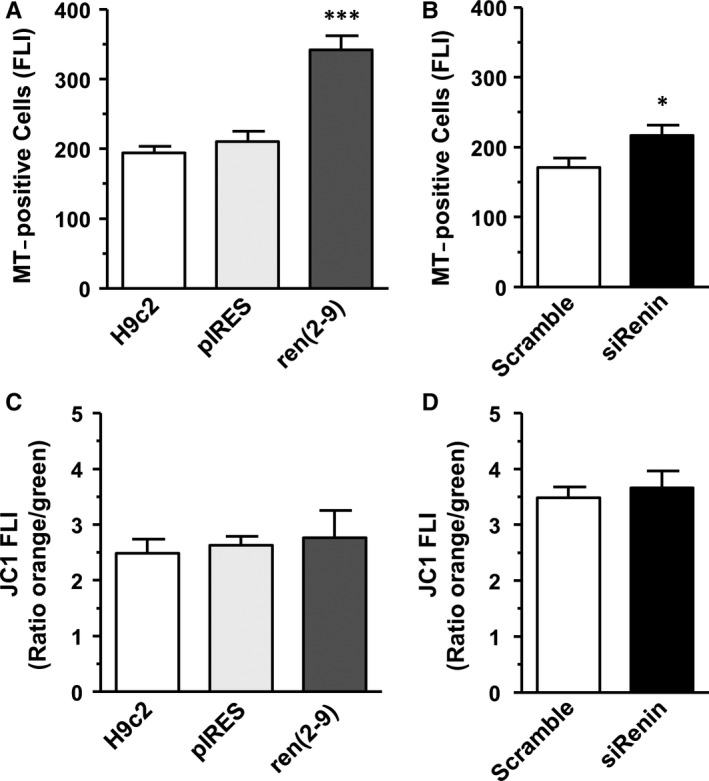
Influence of renin on the mitochondrial membrane potential. Mitochondrial membrane potential was determined as mean fluorescence intensity after addition of MitoTracker red CMXRos (A, B) or JC1 (C, D). Both mitochondrial probes were added either to H9c2, pIRES and ren(2‐9) cells (each n = 10) or scramble controls and renin knockdown cells (n = 10‐14). Fluorescence intensity of the cells was measured by flow cytometry. Data represent mean ± SEM values with ***P < 0.001 or *P < 0.05 ren(2‐9) cells or siRenin‐treated cells to the corresponding controls

Analyses of the fluorescence intensity (FLI) of MitoTracker red CMXRos‐positive cells, an indicator of the mitochondrial membrane potential (∆Ψ_m_) and/or mitochondrial integrity, showed that in ren(2‐9) cells the MitoTracker FLI (341.7 ± 20,9 log U) was significantly enhanced compared to H9c2 and pIRES cells (194.5 ± 9.7 and 210.8 ± 14.4 log U) (Figure 4A). The number of MitoTracker‐positive cells was unchanged between the groups (data not shown). Knockdown of renin resulted in a moderate enhancement of MitoTracker FLI from 170.9 ± 13.2 log U to 216.2 ± 13.2 log U (Figure 4B) and a decrease of MitoTracker‐positive cell number from 96.4 ± 0.5% to 94.1 ± 0.5%.

In addition, we used the fluorescence dye JC1 for detecting the ∆ψ_m_. Here, quantifying the ratio of orange (JC1 aggregates, high ∆Ψ_m_) to green (JC1 monomers, low ∆Ψ_m_) FLI allowed the estimation of ∆Ψ_m_. We did not find any differences between control lines and ren(2‐9) cells as well as between scramble controls and siRNA treated H9c2 cells, indicating an identical level of ∆Ψ_m_ in all cell lines, investigated (Figure 4C, D).

**Figure 5:**
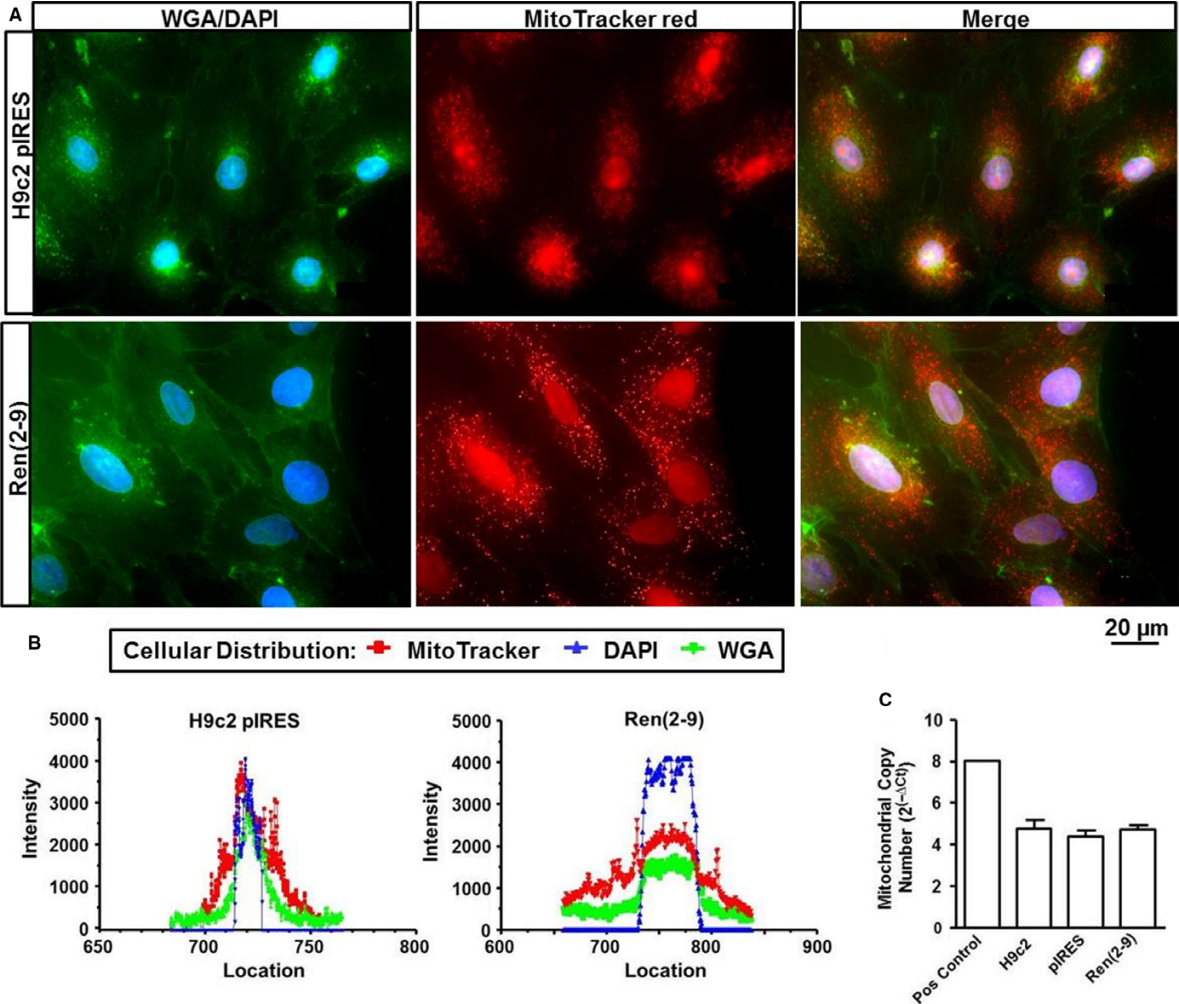
Visualization of the mitochondrial network in H9c2 cell lines. Mitochondrial distribution in pIRES and ren(2‐9) cells was evaluated by the labelling with MitoTracker red CMXRos probe (red) combined with the membrane dye wheat germ agglutinin (WGA, green) and the nuclear dye 4′,6′‐diamidino‐2‐phenylindole (DAPI, blue) (A). Magnification amounted to 100x. Location of mitochondria in relation to the nucleus and the cell membrane was determined by the Line Profile software of the BZ II 9000 microscop (B)

To visualize the mitochondrial network, H9c2 cells were additionally analysed by fluorescence microscopy using MitoTracker red and nonyl‐acridine orange (NAO) labelling (Figure 5). MitoTracker red fluorescence probe clearly demonstrates a cellular distribution of mitochondria in close proximity to the nucleus in control pIRES cells which is confirmed by analysing the so‐called line profile (Figure 5A, B). In contrast, in ren(2‐9) cells lateral expansion of mitochondrial network appears to be more intense. Line profile analyses showed indeed another distribution pattern where mitochondria were more located away from the nucleus towards the cell membrane. Mitochondrial labelling using NAO was complicated by an intensive photobleaching. Therefore, we presented only the data of MitoTracker labelling.

Lastly, the mitochondrial DNA copy number related to the nuclear DNA content was not different between H9c2, pIRES controls, and ren(2‐9)cells, indicating similar mitochondrial contents in the different cell lines (Figure 5C).

### 3.4 Overexpression of ren(2‐9) leads to enhanced extracellular lactate accumulation and LDH activity

**Figure 6:**
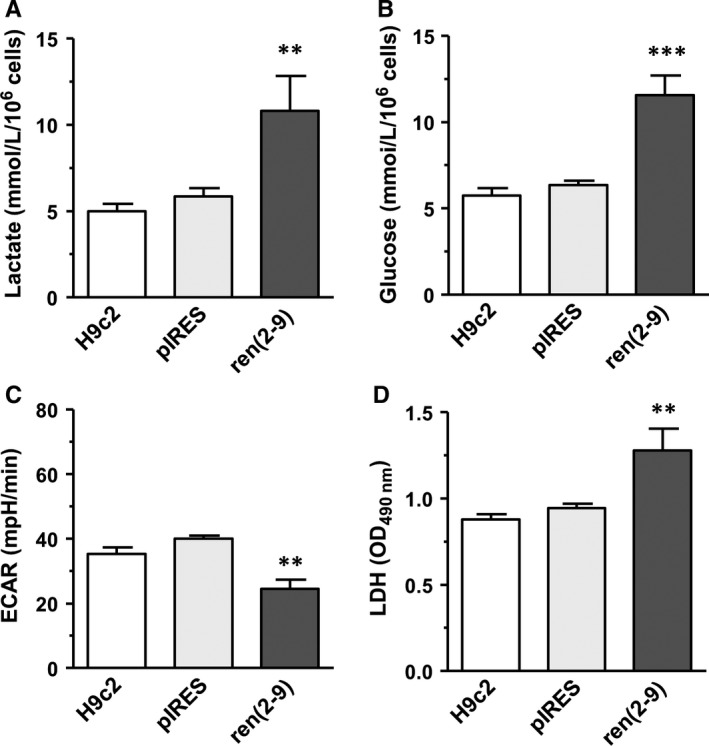
Glycolysis‐related parameters are increased in ren(2‐9) cells. A, Extracellular lactate concentration. B, Glucose uptake. C, Real‐Time measurement of extracellular acidification rate (ECAR, mpH/min) determined by the Seahorse analyser. D, Lactate dehydrogenase (LDH) activity in lysed cells. Data represent mean ± SEM of n = 12 measurements with ***P < 0.01 and **P < 0.01 control H9c2 and pIRES cell lines vs ren(2‐9) cells

**Figure 7:**
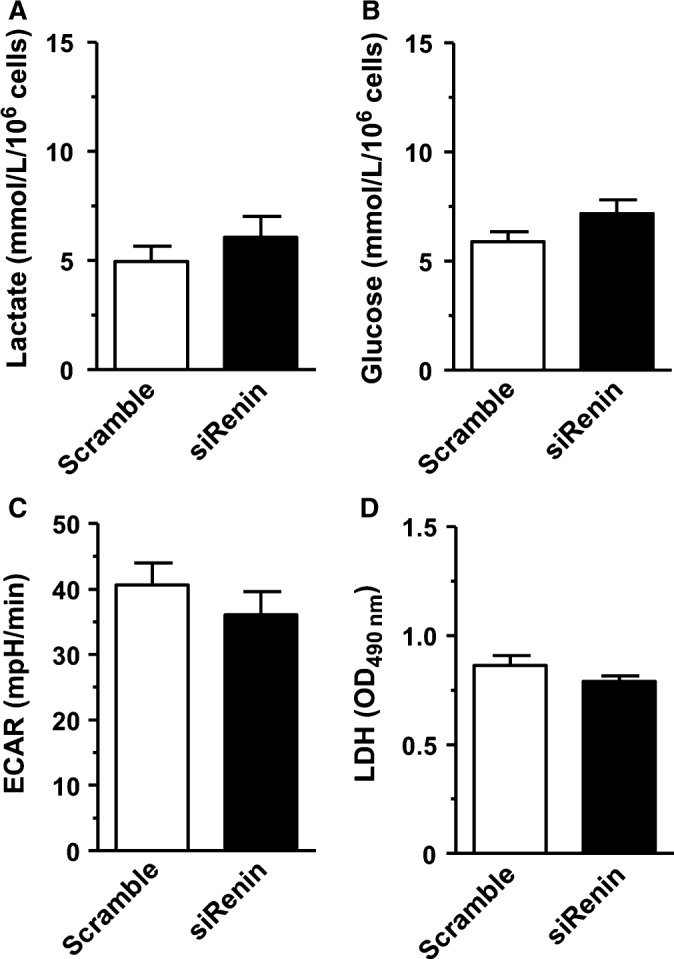
Glycolysis‐related parameters are unchanged in H9c2 cells pretreated by siRNA to renin. A, Extracellular lactate concentration. B, Glucose uptake. C, Real‐Time measurement of extracellular acidification rate (ECAR, mpH/min) determined by the Seahorse analyser. D, Lactate dehydrogenase (LDH) activity in lysed cells. Data represent mean ± SEM of n = 9 values

The decreased ratio between mitochondrial and nonmitochondrial oxygen consumption in ren(2‐9) cells might be due to a switch of energy metabolism from OXPHOS to enhanced glycolysis. In line with this hypothesis, the extracellular lactate accumulation after a 24‐hour culture period was 2‐fold higher in the medium of ren(2‐9) cells (10.8 ± 2.0 mmol/L/10^6^ cells) than that of H9c2 or pIRES controls (5.0 ± 0.4 and 5.8 ± 0.4 mmol/L/10^6^ cells) (Figure 6A). Glucose uptake correlated with the increase of lactate accumulation. Glucose consumption was higher in ren(2‐9) cells (11.6 ± 1.1 mmol/L/10^6^ cells) compared to the controls (H9c2: 5.8 ± 0.4 and pIRES: 6.4 ± 0.3 mmol/L/10^6^ cells) (Figure 6B). Extracellular acidification rate (ECAR) measured simultaneously with the OCR was reduced in ren(2‐9) cells (24.5 ± 2.9 mpH/min) compared to the H9c2 and pIRES controls (35.3 ± 1.9 and 40.1 ± 1.0 mpH/min) (Figure 6C). Lastly, ren(2‐9) cells showed an enhanced lactate dehydrogenase activity (1.3 ± 0.1 OD) compared to H9c2 and pIRES cells (each 0.9 ± 0.03 OD) (Figure 6D). Renin knockdown did not influence any of these metabolic parameters (Figure 7A‐D).

## 4 DISCUSSION

The data presented here indicate that cardiac H9c2 cells overexpressing cyto‐renin exhibit an altered bioenergetic profile, which is based on a switch to more aerobic glycolysis known as Warburg effect. Additionally, these ren(2‐9) cells showed an enhancement of mitochondrial spare capacity which was reduced after renin knockdown. Unfortunately, the Seahorse system did not yield reliable results with primary adult rat cardiomyocytes (see results 3.1). Therefore, we chose the H9c2 cardiomyoblast cell line despite being aware of its limitations with respect to its meaningfulness for the adult heart. However, as the aim of the study was to investigate the so far unknown functions of cyto‐renin, we deem H9c2 cell line to be an appropriate model. Moreover, with respect to the protective effects of cyto‐renin we already demonstrated such effects also in primary adult cardiomyocytes as well as in the isolated perfused heart preparations.^9^

If sufficient substrates such as glucose, pyruvate, and glutamine were available, H9c2 cells carried out mitochondrial oxidative phosphorylation (OXPHOS) but to some extent also glycolysis as demonstrated by the mitochondrial oxygen consumption rate and the extracellular lactate accumulation. Thus, H9c2 cells seem to perform active glycolysis to support mitochondrial function in ATP synthesis.

The measurement of cellular oxygen consuming processes during coordinative inhibition of respiratory chain is the most useful test to estimate mitochondrial function. In ren(2‐9) cells, the activity of nonmitochondrial oxygen consumers amounted to 40% of cellular oxygen uptake and this was twice as high as in control cells. Nonmitochondrial oxygen consumers are, for example, the cytosolic NADPH oxidase, several desaturases and detoxification enzymes^10^ but also extra‐mitochondrial ATPases.^12^ Herst et al^13^ demonstrated that nonmitochondrial respiration can also be linked to cell surface oxygen consumption via trans‐plasma membrane electron transport, leading to enhanced extracellular acidification. The authors hypothesized that the cell surface oxygen consumption indicates the extent of aerobic glycolysis, ie, glycolysis despite the presence of oxygen, known as Warburg effect. In line with this hypothesis, cyto‐renin overexpressing cells exhibit increased extracellular lactate accumulation and cellular lactate dehydrogenase (LDH) activity together with reduced mitochondrial ATP‐linked respiration. Thus, the enhanced glucose uptake could be a mechanism to compensate the limited ATP yield of only 2 ATP per glucose breakdown. All together, these data may indicate a shift from OXPHOS to more aerobic glycolysis, ie, lactate production. However, there are also data disclaiming this assumption. Thus, the extracellular acidification rate (ECAR) was reduced in ren(2‐9) medium despite enhanced lactate accumulation. This can be explained by the fact that besides glycolytic lactate production a number of proton transporters in the plasma membrane as well as the generation of CO_2_ in the tricarboxylic acid cycle take part in the extracellular acidification.^14^, ^15^ Thus, conversion of one glucose molecule to lactate yields two molecules of lactateˉ and two H^+^, while complete breakdown of glucose to CO_2_ yields 6 HCO_3_ˉ and 6 H^+^. Therefore, the ECAR should be three times greater when glucose is converted completely to CO_2_. Because H9c2 cells performed both OXPHOS and glycolysis, the observed acidification rate of H9c2 cells should reflect both proton‐generating pathways. Therefore, in ren(2‐9) cells a preferential execution of aerobic glycolysis could indeed be associated with the observed reduced ECAR.

From a bioenergetic point of view, aerobic glycolysis is inefficient because of its limited ATP yield. Nevertheless, it may provide an advantage for the survival in a hypoxic environment as seen in cancer cells.^16^ Also in neuronal cells, an upregulation of aerobic glycolysis resulted in a decreased mitochondrial respiration and, as a consequence, in the decreased production of reactive oxygen species (ROS) as well as an enhanced resistance to stress factors.^17^ Vice versa, an age‐dependent decrease of aerobic glycolysis was accompanied by an increased mitochondrial respiration and ROS production and led finally to neuronal cell death.^17^ In this sense, in ren(2‐9) cells the switch between mitochondrial OXPHOS and aerobic glycolysis appears to be more balanced to avoid harmful generation of ROS during ischaemia/reperfusion and to maintain a high level of energetic plasticity for surviving. A dysfunctional heart usually lacks this metabolic plasticity.^18^

In most cells, mitochondria operate at a basal level using only a small fraction of the available maximal energetic capability. Maximal oxygen consumption after uncoupling of the respiratory chain by FCCP reflects the maximum activity of electron transport and substrate oxidation.^10^ This parameter is enhanced in ren(2‐9) cells and decreased after renin depletion, indicating an influence of cyto‐renin on the electron transport chain activity and/or the substrate oxidation. Integration of the cell respiratory control ratio_max_ (CRC_max_) affirmed this statement. RCR_max_ which is enhanced in ren(2‐9) cells, too, is sensitive to changes in substrate oxidation and proton leak whereby proton leak was unchanged. The difference between the basal and the maximal respiratory activity represents the spare respiratory capacity (SRC). Thus, SRC indicates how close to its bioenergetics limit a cell is operating. In the heart, it has been shown that the SRC is depleted under severe stress conditions such as high workload during continuous catecholamine infusion, oxidative stress, or ischaemia.^11^, ^19^, ^20^ This decrease is then connected with an increase of cell death. Based on these findings, the decreased SRC as detected in renin knockdown cells could be responsible for the enhanced cell death under basal conditions and after glucose deprivation.^9^ Because, the SRC also represents the amount of oxygen consumption that is available for cells to use under conditions of increased ATP demand or during stress, it should support maintenance of organ function and cellular repair during such conditions. In this context, Dranka et al^21^ hypothesize that a therapeutic increase of SRC could be protective in diseases such as hypertension or atherosclerosis that are associated with increased oxidative stress. Furthermore, it is important to notify that mitochondria in excitable cells, such as cardiomyocytes, are exposed to high fluxes of calcium and other ions, where the proton gradient is utilized to remove toxic calcium concentrations from the cytosol.

Although SRC is a well‐recognized phenomenon, data regarding its regulatory components or factors are limited. One potential source that determines levels of both maximal respiration and SRC is the substrate availability that is synchronized with the activity of electron transport chain.^22^ Our initial investigations clearly show that maximal OCR and spare capacity were decisive influenced by the cell number seeded per well. Because increased cell density is, of course, associated with an increased consumption of substrates ie, glucose as well as oxygen the reduced maximal respiration may be due to technical limitations caused by a reduced flow of oxygen but also to the reduced substrate availability. In this context, Sansbury et al^23^ demonstrated that neonatal cardiomyocytes metabolizing pyruvate had a 4‐fold higher spare capacity and were more resistant to mitochondrial dysfunction induced by 4‐hydroxy‐2‐nonenal than cells using glucose as substrate. The authors speculated that the glucose‐mediated decrease of maximal respiration and spare capacity could be due to a compensatory increase of glycolysis and/or a negative regulation of OXPHOS by glycolytic intermediates. However, in our experiments ren(2‐9) cells showed both an increase of glucose consumption as well as an enhanced spare capacity. Possibly, in H9c2 cardiomyoblasts the increased glycolytic flux could also deliver more pyruvate to mitochondria, thereby increasing the respiratory capacity. A sensitive parameter controlling the activity of the mitochondrial electron transport chain, the respiratory rate and the ATP synthesis is the mitochondrial membrane potential (∆Ψ_m_), ie, the proton‐motive force.^24^ We determined ∆Ψ_m_ by the fluorescent probes JC1 and MitoTracker red CMXRos.^25^, ^26^ Surprisingly, we found different results using these probes. While control‐ and ren(2‐9) cells showed similar ratios of orange/green JC1 fluorescence intensities, indicating similar level of ∆Ψ_m_, the intensity of MitoTracker red fluorescence was significantly enhanced in ren(2‐9) mitochondria. Up to now, the dual‐colour (orange and green) ratiometric assessment of mitochondrial polarization state by JC1 is a more reliable indicator of ∆Ψ_m_ than other mitochondria tracking probes^27^ implicating that the enhanced spare capacity in ren(2‐9) cells did not be due to the ∆Ψ_m_.

Besides ∆Ψ_m_ mitochondrial mass, morphology, or localization can contribute to changes in the fluorescence intensity of such probes. Buckman et al^26^ found that the loading of MitoTracker red is ∆Ψ_m_ dependent but is also influenced by the oxidant status. However, our preliminary data exclude a modified ROS level in ren(2‐9) cells under basal conditions (data not shown). In other studies, enhanced MitoTracker red fluorescence intensity was associated with a hyperfusion of mitochondria^28^ or an increase of mitochondrial mass.^29^ From our analyses, we conclude that the enhanced MitoTracker red FLI in ren(2‐9) cells was not influenced by an altered mitochondrial mass but could be due to a shift of mitochondria away from the nucleus. However, because nonyl‐acridine orange labelling was subjected to a marked quenching effect, additional studies are necessary to verify this observation.

Altogether, our data demonstrate that the protective effects of cytosolic renin on the one side are associated with a shift from OXPHOS to more aerobic glycolysis. This improves the metabolic flexibility and therefore the ability to better cope with fluctuating nutrient and oxygen supplies as observed during ischaemia. On the other side, the increase of spare respiratory capacity could maintain sufficient functional mitochondria to avoid oxidative damage or calcium overload.

It is quite likely that cyto‐renin compensates for high renin states in ischaemic conditions. Indeed, we previously showed that overexpression of secretory renin increased necrosis rate while overexpression of cyto‐renin decreased necrosis rate in the same cell model.^6^ However, in H9c2 cells even in the absence of secretory renin expression we observed markedly protective effects under glucose depletion.^9^ Thus, cyto‐renin may be a promising new target for the prevention of ischaemia‐induced myocardial damage. These findings may be useful for the development of new therapeutic strategies.

## CONFLICT OF INTEREST

The authors declare that they have no conflicts of interest.
